# Adhesion Properties of *Lactobacillus plantarum* Dad-13 and *Lactobacillus plantarum* Mut-7 on Sprague Dawley Rat Intestine

**DOI:** 10.3390/microorganisms9112336

**Published:** 2021-11-11

**Authors:** Arum Darmastuti, Pratama N. Hasan, Rachma Wikandari, Tyas Utami, Endang S. Rahayu, Dian Anggraini Suroto

**Affiliations:** 1 Faculty of Agricultural Technology, Universitas Gadjah Mada, Flora Street No 1 Bulaksumur, Yogyakarta 55281, Indonesia; arum.darmastuti@mail.ugm.ac.id (A.D.); rachma_wikandari@mail.ugm.ac.id (R.W.); tyas_utami@ugm.ac.id (T.U.); 2 Center for Food and Nutrition Studies, Universitas Gadjah Mada, Yogyakarta 55281, Indonesia; pratama.nur.h@mail.ugm.ac.id; 3 University Center of Excellence for Research and Application on Integrated Probiotic Industry, Universitas Gadjah Mada, Yogyakarta 55281, Indonesia

**Keywords:** probiotic, *Lactobacillus plantarum*, hydrophobicity, autoaggregation, adhesion ability

## Abstract

Adhesion capacity is considered one of the selection criteria for probiotic strains. The purpose of this study was to determine the adhesion properties of two candidate probiotics, *Lactobacillus plantarum* Dad-13 and *Lactobacillus plantarum* Mut-7. The evaluation included the hydrophobicity of the cell surface using microbial adhesion to hydrocarbons (MATH), autoaggregation, and the adhesion of *L. plantarum* Dad-13 and *L. plantarum* Mut-7 to the intestinal mucosa of Sprague Dawley rat, followed by genomic analysis of the two *L. plantarum* strains. *L. plantarum* Dad-13 and *L. plantarum* Mut-7 showed a high surface hydrophobicity (78.9% and 83.5%) and medium autoaggregation ability (40.9% and 57.5%, respectively). The exposure of both isolates to the surface of the rat intestine increased the total number of lactic acid bacteria on the colon compartment, from 2.9 log CFU/cm^2^ to 4.4 log CFU/cm^2^ in *L. plantarum* Dad-13 treatment and to 3.86 log CFU/cm^2^ in *L. plantarum* Mut-7 treatment. The results indicate the ability of two *L. plantarum* to attach to the surface of the rat intestine. The number of indigenous *E. coli* in the colon also decreased when the compartment was exposed to *L. plantarum* Dad-13 and Mut-7, from 2.9 log CFU/cm^2^ to 1 log CFU/cm^2^. Genomic analysis revealed that both strains have genes related to adhesion properties that could play an important role in increasing the adherence of probiotics to the intestinal mucosa such as gene encoding fibronectin-binding protein, chaperonin heat shock protein 33 (Hsp33)**,** and genes related to the capsule and cell wall biosynthesis. Based on these findings, we believe that *L. plantarum* Dad-13 and *L. plantarum* Mut-7 have adhesion properties to the intestinal mucosa in the rat intestine model system. The present research will be essential to elucidate the molecular mechanism associated with adhesion in our two probiotic strains.

## 1. Introduction

Probiotics are living microorganisms that, when consumed in sufficient quantities, can provide health benefits [1]. Most probiotic bacteria are lactic acid bacteria (LAB), such as *Lactobacillus* species, *Lactobacillus casei*, *L. paracasei*, and *L. rhamnosus*. *L. plantarum* are the most common probiotic bacteria, representing a potential group of microbes, and have been widely used in various fermented food products [2]. Fermented foods that contain LAB can provide benefits in terms of bodily health when properly consumed. These beneficial effects are associated with the balance of the healthy gut microbiota, as well as the prevention of diarrhea, reduction in cholesterol, regulation of lactose intolerance, and gastrointestinal comfort [3]. Nowadays, products of lactic acid bacteria have been widely applied in the food industry in dairy products, vegetables, meat, wine, etc. [4].

Lactic acid bacteria that can be categorized as probiotics must have several crucial features, including resistance to gastric fluids (low pH), resistance to bile salts, good ability to live in the digestive tract, ability to attach to the intestinal mucosa, and antimicrobial activity against pathogens [3,5]. According to Okochi et al [6], the ability of LAB to attach to the intestinal mucosa can increase the survival rate of LAB in the gastrointestinal tract and enable probiotics to grow. Good adhesion capabilities can protect the intestinal mucosa from pathogenic bacteria [7]. The adhesion properties of LAB are associated with the properties and ability of bacteria to aggregate [8].

The bacterial adhesion mechanism on the gastrointestinal surface involves a nonspecific hydrophobic group interaction and a specific adhesion–receptor interaction. Nonspecific and reversible interactions could occur through involving physicochemical interaction, such as the surface hydrophobicity of LAB cells [9]. On the other hand, specific and irreversible interactions could occur through mediating adhesins, one of the proteins found on the surface of LAB cells, which help the colonization process of the bacterial cell surfaces and involve complementary receptors on host cells [10].

Probiotics capable of forming colonies in the intestinal tract are needed to improve the balance of intestinal microflora [11]. Probiotics must have the ability to adhere to the intestinal cell to increase their chance of survival in the gastrointestinal tract [6,12]. Some probiotic strains can inhibit the adherence of pathogenic bacteria to the intestinal mucosa either by forming a barrier via autoaggregation or by direct coaggregation with the pathogens [13,14,15]. Lactobacilli that have a high autoaggregation ability show a high hydrophobicity [16,17]. It was also reported that the proteins, glycoproteins, and teichoic and lipoteichoic acids on the cell wall surface of bacteria play important roles in the autoaggregation and hydrophobicity of the various strains [18].

Autoaggregation and the hydrophobic properties of the cell surface have been associated with adhesion. To achieve the desired benefits of probiotic bacteria, they must form a sufficiently large biomass through aggregation [19]. A previous study by Nuraida et al. [20] mentioned that the positive hydrophobicity result of LAB indicated its hydrophobic properties. These hydrophobic properties indicate that it may be easier for LAB to attach to the gastrointestinal tract. Microbial adhesion to hydrocarbons (MATH) is the common method that is used to measure the hydrophobicity properties of the surface of lactic acid bacteria. Hydrophobicity is evaluated as the affinity of microorganisms to a solvent, such as hexane, xylene, or toluene [19]. When probiotics adhere to the epithelium, they can function stably in the intestine. Therefore, probiotic functions can be beneficial to the gut and health [21]. As mentioned by Ferreira et al. [15], the autoaggregation ability plays an important role in cell adherence properties, involving the ability of cells to form colonies, which can therefore affect their ability to survive and persist in the gastrointestinal tract. Generally, autoaggregation (%) is classified into three groups: *high* autoaggregation (>70%), *medium* autoaggregation (20–70%), and *low* autoaggregation (<20%) [22]. Both hydrophobicity and autoaggregation are necessary in order for the probiotic to bind better to epithelial cells and thus colonize the gastrointestinal tract [23].

Our laboratory has successfully isolated and characterized indigenous probiotic strains—namely *Lactobacillus plantarum* Dad-13 and *Lactobacillus plantarum* Mut-7. *Lactobacillus plantarum* Dad-13 was isolated from fermented buffalo milk (Dadih) in the West Sumatra region, while *Lactobacillus plantarum* Mut-7 was isolated from fermented dried cassava (Gatot) [24]. Both LABs have potential as good probiotics because they can survive at acidic pHs (pH 2), are resistant to bile salts at levels of up to 3%, and can inhibit pathogenic bacteria such as *Shigella dysenteriae* dky-4, *E. coli* ST, and *E. coli* OK111. However, the adhesion properties of *L. plantarum* Dad-13 and *L. plantarum* Mut-7 have not yet been established.

The adhesion capability of LAB on the surface of the mucosa is necessary in order for LAB to form colonies in the digestive tract so that it can function as a probiotic [25]. The adhesion ability of LAB can be affected by hydrophobicity and the ability of bacteria to aggregate (autoaggregation) [21]. LABs with hydrophobic surface properties find it easier to attach to the intestinal tract. Moreover, lactic acid bacteria that can form aggregates or colonies of the same strain could possibly facilitate the adhesion process in the gastrointestinal tract [26].

In the present study, our objective was to investigate the adhesion properties of *Lactobacillus plantarum* Dad-13 and *L. plantarum* Mut-7 in the rat intestine and identify genes that play a role in the adhesion of LAB in the intestinal tract.

## 2. Materials and Methods

### 2.1. Microorganism and Culture Preparation

This study used 2 isolates of lactic acid bacteria—namely, *Lactobacillus plantarum* Dad-13 (isolated from curd, fermented buffalo milk) and *L. plantarum* Mut-7 (isolated from gatot, fermented cassava)—which were obtained from the Food and Nutrition Culture Collection (FNCC), Center for Food and Nutrition Studies, Universitas Gadjah Mada. 

### 2.2. Preparation of Bacterial Culture

Lactic acid bacteria were grown in MRS (*de Man, Rogosa and Sharpe,* Merck, Darmstadt, Germany) and incubated at a temperature of 37 °C for 24 h before their use.

### 2.3. The Cell Surface Hydrophobicity Assays of LAB

The method used for testing the properties of LAB cell surfaces was microbial adhesion to hydrocarbons (MATH) [19]. The principle of this method is to measure the affinity of the suspension against xylene, which is used as a hydrocarbon solvent. The strain was grown in MRS broth for 18 h at a temperature of 37 °C, then centrifuged. The medium was removed and the cells were washed twice with a buffer solution of magnesium sulfate phosphate (PUM) (composition (g/L): K_2_HPO_4_·3H_2_O: 22.2, KH_2_PO_4_: 7.26. urea: 1.8, MgSO_4_·7H_2_O: 0.2, pH 7.1). The suspension of bacteria formed and then the absorption was measured using a spectrophotometer (Genesys 150, Thermoscientific, Waltham, MA, USA) with a wavelength of 600 nm. A total of 5 mL of bacterial suspension was mixed with 1 mL of xylene. The mixture was incubated at 37 °C for 1 h. The final absorbance measurement was carried out with a wavelength of 600 nm. The suspension affinity against xylene was measured using the following formula:

Surface hydrophobicity (%) = A_0_ − A/A_0_ × 100%.

Description:

A_0_: OD value of 600 nm initial suspension;
A: OD value of 600 nm suspension after mixed hydrocarbons.

### 2.4. LAB Autoaggregation Properties Assays

Autoaggregation assays were performed according to Nuraidaetal [20]. The strain was grown in MRS broth for 18 h at a temperature of 37 °C and then centrifuged. The medium was removed and the cells were washed with phosphate-buffered saline (PBS) twice. The suspension of bacteria formed and the absorption were measured using a spectrophotometer (Genesys 150, Thermoscientific) with a wavelength of 600 nm. The suspension was measured at the 0 and 5th hours. The suspension was incubated for 5 h at a temperature of 37 °C. Absorbance measurement was carried out by taking a suspension of 1 mL and adding it to 3.9 mL of PBS solution. 

The percentages of autoaggregation properties were calculated using the formulas:

Autoaggregation (%) = 1 − (At/A0) × 100

Description:

At = absorbance at t = 5 h;
A0 = absorbance at t = 0 h.

### 2.5. LAB Adhesion Properties Assays on the Rat Intestine

The adhesion properties of two lactobacilli were determined using the method of Nuraida et al [20] with some modifications. LAB isolates were grown in MRS broth for 24 h and then centrifuged at 3500 rpm for 15 min. The cell pellet formed was washed with PBS and resuspended with PBS until a level of 10^6^ CFU/mL was reached. Two compartments of 8-week-old Sprague Dawley rat intestines were used—i.e., the ileum and the colon. The ileum and colon compartments were cut at about 5 cm, opened, and rinsed using a phosphate buffer solution (PBS). A piece of rat intestine was placed in a Petri dish and 10 mL of LAB isolate suspension was added. The Petri dish was incubated at room temperature for 60 min. At the end of the incubation, the rat intestine was removed and washed with PBS, then added to a sterile Petri dish. To measure the amount of LAB attached and the *E. coli* of the surface of the intestine, 1 cm^2^ of the intestine surface was swabbed using a sterile cotton bud. Cells in the cotton bud were suspended in 0.85% NaCl solution and serial dilution was performed. The LAB from the cell suspension was enumerated using MRS agar, while *Escherichia coli* was enumerated using Brilliance *E. coli*/coliform selective medium. The tests were carried out twice.

### 2.6. Genome Sequencing and Analysis

The genome sequencing of the 2 Lactobacillus strains was carried out using the Illumina NovaSeq 6000 sequencing platform. The genome sequence analysis of *L. plantarum* Dad-13 and *L. plantarum* Mut-7 isolates was performed using rapid annotation in the Subsystem Technology (RAST) V2.0 software online.

### 2.7. Statistical Analysis

The data were analyzed using IBM SPSS Statistics version 20. The triplicate results of cell surface hydrophobicity and autoaggregation assays were analyzed using t-tests. The intestines of the two rats were used for testing the adhesion ability and the amount of indigenous *Escherichia coli* present, in which each test was performed twice. A one-way ANOVA followed by Duncan’s test was performed to identify the difference in the results.

## 3. Results and Discussion

### 3.1. Properties of Surface of LAB Cells

The results of the tests of cell surface properties in the isolates *L. plantarum* Dad-13 and *L. plantarum* Mut-7 are shown in Table 1. According to the table below, both isolates had positive results for cell surface hydrophobicity.

In this study, xylene was used as the solvent. Having a low affinity (negative) for xylene indicates that the surface of bacterial cells is hydrophilic while having a high affinity (positive) for xylene indicates that bacterial cells are hydrophobic [20]. Gomaa et al. [27] reported that effective hydrophobicity was observed when xylene was used as a solvent in all the lactobacilli strains. 

In the present study, the hydrophobicity of the cell surface of *L. plantarum* Dad-13 and *L. plantarum* Mut-7 was evaluated and both strains showed a variable degree of hydrophobicity around 78.9% and 83.5%, respectively [28]. *L. plantarum* of six strains showed a good level of hydrophobicity and *L. plantarum* RYPR1 had the highest affinity for xylene (79.13%) out of all the isolates. A previous study showed that several strains of *L. plantarum* had high hydrophobicity values (>60%), with *L. plantarum* IFPL189, IFPL124, IFPL33, and IFPL207 having the highest affinities to xylene with values greater than 90% for the hydrophobicity of the cell surface [12]. A similar study by Handa et al. [29] reported that *L. plantarum* F22 had a high value of cell hydrophobicity of around 50.8%.

Based on several studies, the hydrophobicity levels varied among the strains. The diversity of hydrophobicity based on the MATH method was caused by the influence of the variety of strains, the duration of the cultivation time, the cultivation medium used, the presence of acids, and the type of solvent used [19]. Hydrophobicity can differ between strains of species and changes with a change in the strain, suspension media composition, age of the bacteria, and structure of the cell surface [18]. The hydrophobicity of the probiotic cell surface increases when lactose is used as the sole carbon source [30]. On the contrary, the presence of elaidic acid could probably decrease the hydrophobicity level of lactobacilli [31]. 

The results of the autoaggregation properties of *L. plantarum* Dad-13 and *L. plantarum* Mut-7 isolates are shown in Table 1. Based on the test, it was found that the value of autoaggregation (%) in the *L. plantarum* Dad-13 isolate was around 40.9% and that in *L. plantarum* Mut-7 was 57.5%. The ability of autoaggregation enabled lactic acid bacteria to form aggregates and colonies that would be necessary for probiotic functions. This could allow one to attach and compete with the pathogenic bacteria that exist on the surface of the intestinal mucosa [12]. Both *L. plantarum* Dad-13 and *L. plantarum* Mut-7 are classified as having medium autoaggregation according to Rahman et al [22]. 

According to the results found by García-Cayuela et al. [12] for several varieties of *Lactobacillus plantarum*, selected *Lactobacilli* had high autoaggregation abilities of around 29.3–59.5%. Another study carried out by Handa et al. [29] concluded that the autoaggregation percentage increased until the 5 h time point and in the final hour showed a high autoaggregation ability (79.5%). Based on the results of Tuo et al. [21], no significant correlation was found between the cell surface hydrophobicity and autoaggregation among *Lactobacilli* strains.

### 3.2. Properties of LAB Adhesion to the Rat Intestine

A test was used to determine the level of LAB that attached to the surface of the rat intestine and the results are shown in Table 2. The initial quantities of *L. plantarum* Dad-13 and *L. plantarum* Mut-7 isolates were 10^6^–10^7^ CFU/mL. Based on the results, *L. plantarum* Dad-13 and *L. plantarum* Mut-7 isolates were capable of attaching to the colon; however, neither attached in the ileum compartment.

According to Wang et al. [32] among several strains of *Lactobacillus,* most were able to adhere well to Caco-2 and IEC-6 cells. The highest capacity of adherence was observed with commercial *L. acidophilus* L050103, *L. plantarum* L2, *L. plantarum* L8, and *L. plantarum* L9. Previous studies concluded that among several strains of *L. reuteri*, *L. casei*, *L. brevis,* and *L. paracasei*, the results showed that all tested strains were able to adhere well to the mixed cell cultures of Caco-2 cells and mucus-secreting HT-29-MTX [19,33]. Furthermore, as shown by Nuraida et al. (2012), *Lactobacillus sp.* and *L. rhamnosus* had the ability to adhere to the surface of the rat intestine.

Several studies have tested the adhesion ability of probiotic strains; however, their findings are hardly comparable due to differences in the tissue model used. Different results from previous studies might occur due to the difference in the substrate used in the adhesion properties test. Substrate cells have different physicochemical and receptor properties that are influenced by the type of compound used, structure, and conformation [9].

The results of this study showed that *L. plantarum* Dad-13 and *L. plantarum* Mut-7 were able to decrease the amount of indigenous *E. coli* in the colon (Table 3), which indicated that LAB was able to replace indigenous *E.* coli. The replacement of indigenous *E. coli* was probably caused by the attachment of *L. plantarum* Dad-13 and *L. plantarum* Mut-7 to the colon, according to the results of Table 2. Furthermore, there was a significant difference in the number of indigenous *E. coli* in the ileum and colon compartments.

Two compartments of rat intestine were used in the present study: the ileum and colon. Although both compartments have superficial similarities, the ileum and colon have considerable differences in their physiology [34]. The thickness of the mucus layer tends to increase by about 30 to 300 µm from the small intestine to the large intestine, and this might provide a more extensive mucosal habitat for lactic acid bacteria in the colon compared with the small intestine parts [35,36].

Some previous studies have shown that there was no correlation between the surface hydrophobicity of Lactobacillus and its ability to adhere to the intestinal mucosa [37,38,39]. However, some studies have reported a correlation between hydrophobicity and adhesion capacity. The higher the surface hydrophobicity value is, the higher the ability of bacterial cells to adhere to the intestinal mucosa is [10,40].

The correlation between hydrophobicity and adhesion capability might occur because there are nonspecific interactions on the surface of the bacterial cells with the intestinal mucosa. The distance between the two surfaces of the bacterial cells and the intestinal mucosa causes an attractive force that is involved in hydrophobic interactions, resulting in reversible binding, and this is possible because the gastrointestinal mucosa is mostly hydrophobic due to the presence of mucus on its surface [9]. Although the detailed mechanism explaining the correlation between surface hydrophobicity of bacterial cells and adhesion to the intestinal mucosa is not clear, hydrophobicity and autoaggregation testing could be used as indicators in the initial screening to identify the potential adherence of lactic acid bacteria [10]. The present finding suggests that the adhesion ability test of the probiotic may provide important screening tools to determine the feasibility of lactic acid bacteria as probiotics.

### 3.3. Genomic Analysis

**Figure 1:**
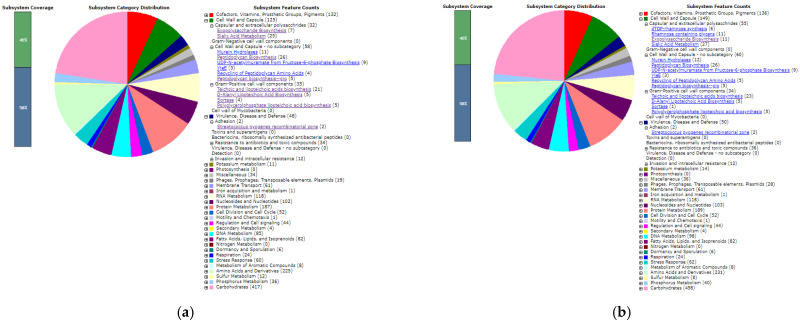
(**a**) Distribution of the *L. plantarum* Dad-13 subsystem of RAST annotation; (**b**) distribution of the L. *plantarum* Mut-7 subsystem of RAST annotation.

The results of genome annotations using RAST are shown in Figure 1a,b. Both figures showed the distribution of the subsystems in each isolate. In the bar chart, it appears that the 42% isolate coverage subsystem shows the entire subsystem working in physiological isolates. In the category of virulence, disease, and defense, the existence of subcategory adhesion includes the recombinatorial zone of *Streptococcus*. In this subsystem, it is evident that both isolates have gene specifications and functional functions, as shown in Table 4.

*L. plantarum* Dad-13 and *L. plantarum* Mut-7 have important properties as probiotics, with genes that regulate adhesion and self-defense against antimicrobials. They contain genes that encode fibronectin-binding proteins (ptrF/polymerase I and transcript release factor) and chaperonin (hsp33/heat shock protein 33), which regulate the adhesion of bacteria (Table 5).

The results of the genome annotations based on RAST showed the presence of genes related to the adhesion properties of LAB—namely, polymerase I, transcript release factor (ptrF), and heat shock protein 33 (hsp33). Both genes were known to have a 100% identification to other species present in Lactobacillaceae based on the protein BLAST analysis. The PtrF gene encodes protein F, which is a bacterial surface protein that binds fibronectin at a high affinity. This gene produces a functional fibronectin-binding protein. The fibronectin-binding protein is a protein that can initiate the invasion process of microbes in the endothelium and is able to form a bridge between bacterial proteins that bind to fibronectin and host cell receptors [41,42].

Another gene that possibly plays a role in bacterial adhesion is hsp33. This gene is known as a new primary heat shock protein that has chaperone activity in addition to other variants such as hsp60, hsp70, hsp90, hsp100, and small heat shock proteins [43]. Hsp33 has been identified as a redox-sensitive chaperone that has functions in protecting unfolded proteins from aggregation in bacteria and is activated when oxidative stress and the unfolding condition coincide [44,45]. Furthermore, this heat shock protein could adhere to the host and microbial cell surface; therefore, it might be able to facilitate the easy attachment and colonization of bacteria [46]. In probiotic or lactic acid bacteria, attachment and colonization could be improved by the expression of a molecular chaperone such as heat shock protein and other co-chaperones [47]. However, the mechanism by which hsp33 contributes to adhesion to the cell host remains unclear.

Based on the RAST annotation, both *L. plantarum* Dad-13 and *L. plantarum* Mut-7 have no genes associated with hemolysin, lipids, and proteins that cause the lysis of red blood cells by disrupting the cell membrane, as one of the virulence factors. According to Rahayu et al. [48]), the consumption of *L. plantarum* Dad-13 does not negatively affect general health, organ weight, leukocyte profile, glutamic-oxaloacetic transaminase (GOT) activity, plasma malondialdehyde (MDA) concentration, or intestinal morphology in Sprague Dawley rats. *Lactobacillus plantarum* Dad-13 does not translocate in the organs and blood. Furthermore, Ikhsani et al. [49] reported that high-dose supplementation with *L. plantarum* Mut-7 does not have detrimental effects on general health, organ weight, hematology, and histological parameters in rats. Further, the bacterial translocation of *L. plantarum* Mut-7 was not detected in the blood or organs. Therefore, based on these findings, it was suggested that both probiotic bacteria are safe for consumption.

Previous studies conducted by Tropcheva et al. [50] on the adhesion ability of *Lactobacillus plantarum* AC131 have revealed the presence of the mucus adhesion protein (MapA), mucus-binding protein (MUB), and Tu elongation factor (EF-Tu) genes as a genetic determinant of adhesion factors in *L. plantarum* AC131. MapA is known as a cell surface protein that has an affinity for molecules in the host’s gastrointestinal tract [36]. In addition, MUB has been designated as a member of the mucin-binding protein (MucBP) family, which contains proteins that play an important role in the establishment of lactic acid bacteria and host interactions in the gut [51]. EF-Tu was found to be a vital chaperone substrate for hsp33 because heat shock protein might prevent the occurrence of the degradation of oxidative proteins [52].

Based on the RAST annotation in Figure 1, it is known that the probiotic bacteria *L. plantarum* Dad-13 and *L. plantarum* Mut-7 have genes that are responsible for the formation of the main components of bacterial cell walls. The *Lactobacillus plantarum* Dad-13 and *L. plantarum* Mut-7 bacteria are classified as Gram-positive bacteria. Gram-positive bacteria have different cell wall components from Gram-negative bacteria. Gram-positive bacteria do not have an outer membrane and have a thicker peptidoglycan layer than Gram-negative bacteria. The main components of the cell walls of Gram-positive bacteria include peptidoglycan, polysaccharides, and teichoic acid [53]. According to several previous studies, cell wall components in bacteria play an important role in the attachment of bacteria to host cells. Table 6 and Table 7 show the types of genes that play a role in the formation of cell walls of the probiotic bacteria *L. plantarum* Dad-13 and *L. plantarum* Mut-7.

Peptidoglycan is the main component of the cell wall of Gram-positive bacteria [54]. Peptidoglycan is a crosslinked matrix of linear carbohydrate (glycan) chains that are connected to each other through covalent bonds between attached peptides [55]. Peptide bonds consist of a matrix of direct and indirect crosslinked that differ between species. Short bonds of one or more amino acids could produce a three-dimensional structure that envelops the cell and functions to maintain the integrity of the bacterial cell wall [54]. Several roles of peptidoglycan in the metabolism of lactic acid bacteria include maintaining resistance to lysozyme, inhibiting autolysis, and acting as a growth factor. The presence of the peptidoglycan [56,57] component in the cell wall of lactic acid bacteria or probiotic bacteria allows it to play an active role in maintaining the immune balance of the gut microbiota in the intestine, and it has a protective role in inflammatory conditions [58]. In addition, one of the components of peptidoglycan—namely, peptidoglycan hydrolase (PGH)—is known to play a role in helping bacteria to survive and live in intestinal epithelial cells through the mechanism of the epidermal growth factor receptor [59].

Teichoic acid is a phosphate-rich cell surface glycopolymer that is widely found in the cell wall components of Gram-positive bacteria [55]. There are two types of teichoic acid: lipoteichoic acid and wall teichoic acid. Lipoteichoic acids adhere to the plasma membrane and stick to the peptidoglycan layer, while teichoic acid from the wall adheres to the peptidoglycan layer and extends to the outside of the bacterial cell wall [53]. Teichoic acid plays a role in protecting cells from cell damage through autolytic activity, cell division, resistance to heat stress, and low osmolarity [60]. In interactions with receptors, teichoic acid is known to act as an intermediary for the attachment of bacterial cells to epithelial and endothelial cells [61]. The presence of teichoic acid and D-alanyl ester affects the interaction of bacteria with various other cell surfaces. A previous study showed that a deficiency of teichoic acid can cause a decrease in the ability of bacterial cells to adhere to host cells. A decrease in attachment ability can increase the negative charge on the surface of bacterial cells, resulting in an increase in repulsion force between the bacterial cell surface and epithelial cells [55,62].

Polysaccharides are one of the components forming the cell walls of Gram-positive bacteria, in addition to peptidoglycan and teichoic acid. Polysaccharides can be classified into three types; (1) exopolysaccharide (EPS), which is present on the surface of microbial cells; (2) capsular polysaccharide (CPS), which is permanently attached to the cell and forms a shield around the bacteria; (3) cell wall polysaccharide (WPS), which covalently or noncovalently binds to the cell wall but does not form a capsule [54]. Polysaccharides in the cell wall contribute to cell division and morphology, act as bacteriophage receptors, offer protection against phagocytosis, and have immunosuppressive functions [63]. Exopolysaccharides found on the surface of bacterial cells play an active role in attachment to the surface of other microorganisms and the formation of biofilms, as well as mediating interactions between other microorganisms and host cells [64].

Cell wall proteins are proteins that are found on the surface of bacterial cells. Approximately 5–10% of bacterial proteins derived from synthesis in the cytoplasm are released outside of the cytoplasmic membrane [65]. Cell surface proteins facilitate the microbial colonization of the mucosa and survival in the gastrointestinal tract in the process of interaction with host cells. The surface protein found on the cell surface of lactic acid bacteria is the mucus-binding protein (MucBP), which plays an important role in the ability of bacteria to attach to the mucosal layer in intestinal epithelial cells [66].

## 4. Conclusions

Both tested strains of *L. plantarum* Dad-13 and *L. plantarum* Mut-7 showed a high cell surface hydrophobicity and a medium autoaggregation capacity. Both strains showed adhesion ability in the colon compartment of the rat intestine, as evidenced by the increasing number of lactic acid bacteria and the low number of *E. coli* present. Further, *Lactobacillus plantarum* Dad-13 and *L. plantarum* Mut-7 featured genes related to adhesion properties that might play important roles in increasing the adherence of probiotics to the intestinal mucosa. Whole-genome sequencing has paved the way to defining genes associated with bacterial adhesion. Future studies are needed to identify the remaining attributes related to genes responsible for adhesion in bacteria.

## Figures and Tables

**Table 1:** Hydrophobicity and autoaggregation (%) of isolate strains.

	Isolates Name	
	*L. plantarum* Dad-13	*L. plantarum* Mut-7
Hydrophobicity (%)	78.9 ± 5.9 ^a^	83.5 ± 5.7 ^a^
Autoaggregation (%)	40.9 ± 2.1 ^a^	57.5 ± 5.5 ^a^

^a,b^ Similar letters in the table indicate no significant difference, while different letters indicate a significant difference (*p* < 0.05).

**Table 2:** The number of LAB found after *L. plantarum* Dad-13 and *L. plantarum* Mut-7 were added with a 60 min incubation.

Compartment	Number of LAB (log CFU/cm^2^)		
	PBS (No Addition of LAB)	*L. plantarum* Dad-13	*L. plantarum* Mut-7
Ileum	3.02 ± 0.03 ^a^	3.5 ± 0.4 ^a^	3.6 ± 0.06 ^a^
Colon	2.9 ± 0.08 ^a^	4.4 ± 0.002 ^b^	3.86 ± 0.56 ^b^

^a,b^ Similar small letters in the same row indicate no significant differences, while different letters indicate significant differences (*p* < 0.05).

**Table 3:** The amount of indigenous *E. coli* present after *L. plantarum* Dad-13 and *L. plantarum* Mut-7 were added with 60 min of incubation.

Comparison	Number of Indigenous *E. coli* (log CFU/cm^2^)		
	PBS (No Addition of LAB)	*L. plantarum* Dad-13	*L. plantarum* Mut-7
Ileum	2.9 ± 0.15 ^Aa^	2.42 ± 0.73 ^Aa^	2.54 ± 0.48 ^Aa^
Colon	2.9 ± 0.52 ^Ab^	1.0 ± 0.00 ^Ba^	1.0 ± 0.00 ^Ba^

^A,B^ Similar capital letters in the same column indicate that there are no significant differences, while different letters indicate significant differences (*p* < 0.05). ^a,b^ Small letters in the same row indicate no significant differences, while different letters indicate significant differences (*p* < 0.05).

**Table 4:** Genes related to the adhesion of *L. plantarum* Dad-13 based on RAST.

Category	Subcategory	Subsystem	Role	Abbreviations	Per. Identifier	Scientific Name
Virulence, Disease, and Defense	Adhesion	Recombination zone of Streptococcus pyogenes	Fibronectin-binding protein	PtrF	100%	Lactobacillaceae
Virulence, Disease, and Defense	Adhesion	Recombination zone of Streptococcus pyogenes	Chaperonin (heat shock protein 33)	hsp33	100%	Lactobacillaceae

**Table 5:** Genes related to the adhesion of *L. plantarum* Mut-7 based on RAST.

Category	Subcategory	Subsystem	Role	Abbreviations	Per. Identifier	Scientific Name
Virulence, Disease, and Defense	Adhesion	Recombination zone of Streptococcus pyogenes	Fibronectin-binding protein	PtrF	100%	Lactobacillaceae
Virulence, Disease, and Defense	Adhesion	Recombination zone of Streptococcus pyogenes	Chaperonin (heat shock protein 33)	hsp33	100%	Lactobacillaceae

**Table 6:** Genes related to the cell wall formation of probiotic bacteria *L. plantarum* Dad-13.

Category	Subcategory	Subsystem	Role	Abbreviations
Cell wall and capsule	Capsular and extracellular polysaccharides	Exopolysaccharide biosynthesis	Transcriptional activator of exopolysaccharide biosynthesis	EpsA
			Exopolysaccharide biosynthesis glycosyltransferase	EpsF
			Acetyltransferase of exopolysaccharide biosynthesis	EpsH
			Capsular polysaccharide synthesis enzyme	CpsA, CpsB, CpsC, CpsD, CpsH
Cell wall and capsule	Cell wall and capsule—no subcategory	Peptidoglycan biosynthesis	Monofunctional biosynthetic peptidoglycan transglycosylase	MG
			Cell division protein FtsI (Peptidoglycan synthetase)	FtsI
			D-alanine ligase	ddlB
Cell wall and capsule	Gram-positive cell wall components	Biosynthesis of teichoic and lipoteichoic acids	Teichoic acid biosynthesis protein	TBP
			Regulation of D-alanyl-lipoteichoic acid biosynthesis	DltR, DltS

**Table 7:** Genes related to the cell wall formation of probiotic bacteria *L. plantarum* Mut-7.

Category	Subcategory	Subsystem	Role	Abbrev.
Cell wall and capsule	Capsular and extracellular polysaccharides	Exopolysaccharide biosynthesis	Transcriptional activator of exopolysaccharide biosynthesis	EpsA
			Exopolysaccharide biosynthesis glycosyltransferase	EpsF
			Acetyltransferase of exopolysaccharide biosynthesis	EpsH
			Capsular polysaccharide synthesis enzyme	CpsA, CpsB, CpsC, CpsD, CpsH
			Capsule polysaccharide export protein	Kps
			Glucose-1-phosphate thymidylyltransferase	RfbA
Cell wall and capsule	Cell wall and capsule—no subcategory	Peptidoglycan Biosynthesis	Monofunctional biosynthetic peptidoglycan transglycosylase	MG
			Cell division protein FtsI (Peptidoglycan synthetase)	FtsI
			D-alanine ligase	ddlB
Cell wall and capsule	Gram-positive cell wall components	Biosynthesis of teichoic and lipoteichoic acids	Teichoic acid biosynthesis protein	TBP
			Regulation of D-alanyl-lipoteichoic acid biosynthesis	DltR, DltS

## Data Availability

All data presented in this study are available in the article.
